# From Eye Care to Hair Growth: Bimatoprost

**DOI:** 10.3390/ph17050561

**Published:** 2024-04-27

**Authors:** Marco Zeppieri, Caterina Gagliano, Leopoldo Spadea, Carlo Salati, Ekele Caleb Chukwuyem, Ehimare Samuel Enaholo, Fabiana D’Esposito, Mutali Musa

**Affiliations:** 1 Department of Ophthalmology, University Hospital of Udine, p.le S. Maria della Misericordia 15, 33100 Udine, Italy; 2 Department of Medicine and Surgery, University of Enna “Kore”, Piazza dell’Università, 94100 Enna, Italy; 3 Eye Clinic, Catania University, San Marco Hospital, Viale Carlo Azeglio Ciampi, 95121 Catania, Italy; 4 Eye Clinic, Policlinico Umberto I, “ Sapienza” University of Rome, 00142 Rome, Italy; 5 Centre for Sight Africa, Nkpor, Onitsha 434112, Nigeria; 6 Imperial College Ophthalmic Research Group (ICORG) Unit, Imperial College, London NW1 5QH, UK; 7 Department of Optometry, University of Benin, Benin City 300238, Nigeria; mutali.musa@uniben.edu

**Keywords:** bimatoprost, glaucoma, alopecia, hypotrichosis, dermatology

## Abstract

Background: Bimatoprost has emerged as a significant medication in the field of medicine over the past several decades, with diverse applications in ophthalmology, dermatology, and beyond. Originally developed as an ocular hypotensive agent, it has proven highly effective in treating glaucoma and ocular hypertension. Its ability to reduce intraocular pressure has established it as a first-line treatment option, improving management and preventing vision loss. In dermatology, bimatoprost has shown promising results in the promotion of hair growth, particularly in the treatment of alopecia and hypotrichosis. Its mechanism of action, stimulating the hair cycle and prolonging the growth phase, has led to the development of bimatoprost-containing solutions for enhancing eyelash growth. Aim: The aim of our review is to provide a brief description, overview, and studies in the current literature regarding the versatile clinical use of bimatoprost in recent years. This can help clinicians determine the most suitable individualized therapy to meet the needs of each patient. Methods: Our methods involve a comprehensive review of the latest advancements reported in the literature in bimatoprost formulations, which range from traditional eye drops to sustained-release implants. These innovations offer extended drug delivery, enhance patient compliance, and minimize side effects. Results: The vast literature published on PubMed has confirmed the clinical usefulness of bimatoprost in lowering intraocular pressure and in managing patients with glaucoma. Numerous studies have shown promising results in dermatology and esthetics in promoting hair growth, particularly in treating alopecia and hypotrichosis. Its mechanism of action involves stimulating the hair cycle and prolonging the growth phase, leading to the development of solutions that enhance eyelash growth. The global use of bimatoprost has expanded significantly, with applications growing beyond its initial indications. Ongoing research is exploring its potential in glaucoma surgery, neuroprotection, and cosmetic procedures. Conclusions: Bimatoprost has shown immense potential for addressing a wide range of therapeutic needs through various formulations and advancements. Promising future perspectives include the exploration of novel delivery systems such as contact lenses and microneedles to further enhance drug efficacy and patient comfort. Ongoing research and future perspectives continue to shape its role in medicine, promising further advancements and improved patient outcomes.

## 1. Introduction

**Figure 1:**
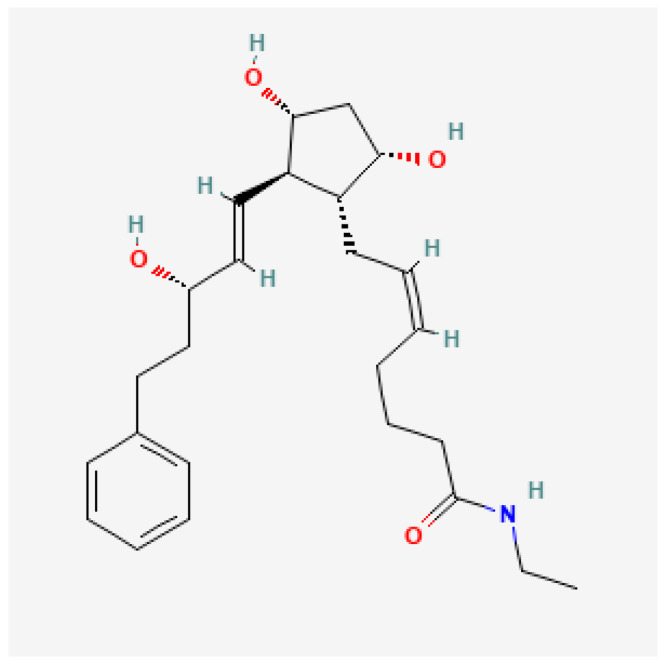
Chemical structure of the bimatoprost molecule.

Bimatoprost has emerged as an essential medication in the field of medicine over the past two decades, with diverse applications in ophthalmology, dermatology, and beyond [1]. It was first approved for use in 2001 [2] and is still extensively researched to understand its long-term prospects and interactions [3]. Bimatoprost amide is classified as a prostaglandin analogue (PGF2α) [4,5] and can be derived from dichloro-containing bicyclic ketone-6a [6]. Initially developed as an ocular hypotensive agent, bimatoprost has proven to be highly effective in the treatment of glaucoma [7,8] and for its neuroprotective properties [9,10,11]. It is a small synthetic molecule and its patent is currently owned by Allergan which is headquartered in Ireland [10]. Its molecular structure is shown in Figure 1 below [10].

Bimatoprost is rapidly hydrolyzed to bimatoprost free acid in the eye which in turn activates its intraocular pressure (IOP) reduction mechanism [12]. With respect to its mechanism of action, bimatoprost closely mimics the prostamides in up-stimulating the rate of aqueous humor flow. Its ocular hypotensive action is due primarily to a 26% reduction in the tonographic resistance to outflow via the trabecular meshwork [13]. Its pharmacokinetics reveal rapid absorption following ocular administration, with peak plasma concentrations typically attained within one to two hours post-application. This rapid absorption is facilitated by the highly vascularized conjunctiva, allowing bimatoprost to enter the systemic circulation swiftly [14]. Once absorbed, bimatoprost undergoes metabolism primarily in the liver via oxidation, hydrolysis, and conjugation processes, ultimately forming inactive metabolites that are excreted primarily through renal elimination. The elimination half-life ranges from approximately 45 min to 4 h, indicative of its relatively short duration of action. Pharmacodynamically, bimatoprost acts by increasing the outflow of aqueous humor from the eye, thereby reducing intraocular pressure (IOP). It achieves this by binding to and activating prostaglandin receptors (specifically, the prostamide receptor) in the ciliary muscle of the eye [13]. Activation of these receptors leads to relaxation of the ciliary muscle and enlargement of the trabecular meshwork spaces, facilitating increased drainage of aqueous humor from the eye. Additionally, bimatoprost may also enhance uveoscleral outflow, further contributing to its IOP-lowering effects [14]. By modulating these physiological pathways, bimatoprost effectively mitigates the risk of optic nerve damage and visual field loss associated with elevated IOP, making it a cornerstone in the management of glaucoma and ocular hypertension.

Thus, bimatoprost enhances the pressure-sensitive outflow pathway [13,14]. Bimatoprost use is reportedly a viable temporary protective measure in cases of non-inflammatory angle closure glaucoma [15]. Glaucoma, often referred to as the “silent thief of sight”, is a group of eye conditions that damage the optic nerve, leading to gradual vision loss and potentially blindness if left untreated. This disease typically develops when fluid pressure within the eye, known as intraocular pressure, increases, causing damage to the optic nerve fibers responsible for transmitting visual information to the brain. The onset of glaucoma is insidious, often progressing without noticeable symptoms until significant vision loss occurs [16]. While there is no cure for glaucoma, early detection through regular eye exams and timely treatment, such as medication, laser therapy, or surgery, can help slow its progression and preserve remaining vision. Glaucoma underscores the importance of proactive eye care and underscores the necessity of regular eye check-ups to maintain healthy vision.

Shen et al. measured bimatoprost presence in quantitative amounts up to one hour post-application, and bimatoprost acid up to seven hours after application in the aqueous and ciliary processes [17]. Heo et al., among other authors, suggested that bimatoprost achieves its ocular hypotensive effects by increasing the activity of matrix metalloproteinases, specifically MMPs-1,3,9 and TIMP-2,4 [18,19]. Stamer et al. suggested that the cellular basis for the antiglaucoma effects of bimatoprost can also be viewed from its ability to decrease cellular contractility of the trabecular meshwork, canal of Schlemm, and ciliary smooth muscle [20]. Its ability to reduce intraocular pressure has made it a first-line treatment option for these conditions [21], offering patients improved management and prevention of vision loss. Bimatoprost is also a viable option for individuals with reduced response to latanoprost due to the activity of Allele C in the rs3753380 receptor [22]. Multiple clinical trials have been concluded and have unanimously found bimatoprost to be a viable drug for intraocular pressure reduction [23].

We aim to provide a brief description and overview of studies in the current literature regarding the versatile clinical use of bimatoprost in the past two decades. Descriptive headings have been used to summarize the specific issues regarding bimatoprost in this paper. The purpose of this review is not to present a prospective clinical study, however, but to provide a quick overlook of the versatile uses of this substance in different fields of medicine, and the evolution of how things have changed regarding its posology and usage since 2000.

## 2. Methods 

**Figure 2:**
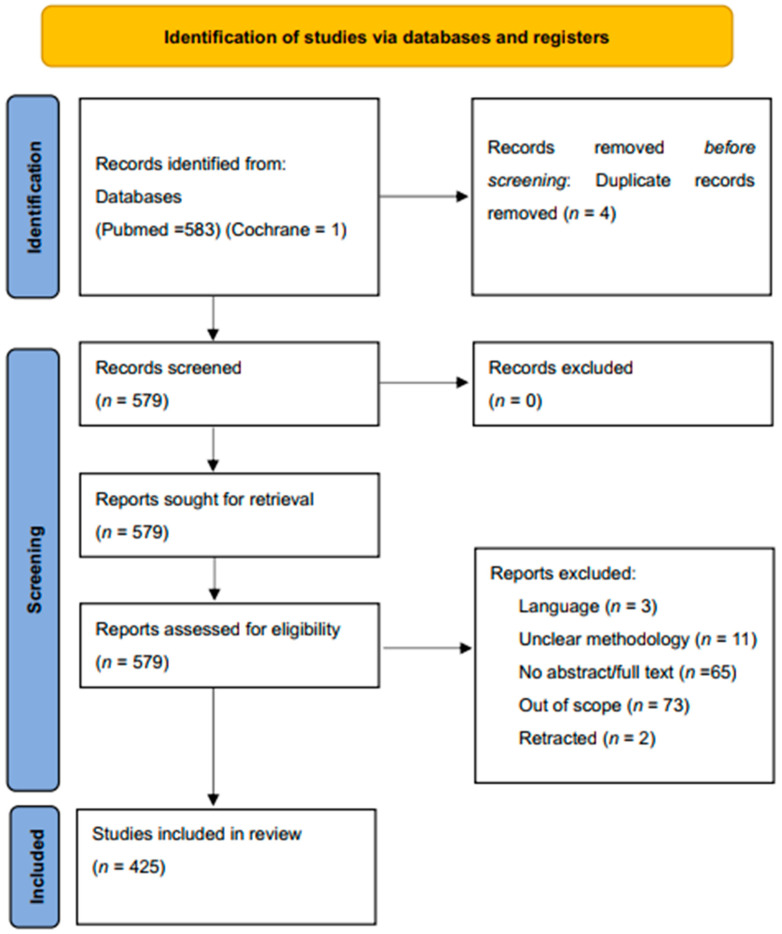
PRISMA flowchart showing selection of articles used in this study.

The authors developed inclusion criteria comprising published articles from 2010 and newer ones available in PubMed and Cochrane libraries. Full-length publications, review papers, and case reports were considered for this research using the search string “(“bimatoprost”[MeSH Terms] OR “bimatoprost”[All Fields]) AND (2010:2023[pdat])”. Papers that were not in the English language, outside the scope of this review, and duplicates were excluded. A total of 583 records were returned for PubMed while only one record was returned for Cochrane Library. A minimum of two of the authors reviewed each study for relevance and assigned ratings to them. Figure 2 shows the flow chart according to the PRISMA [24] and the details regarding the selection.

## 3. Results

**Figure 3:**
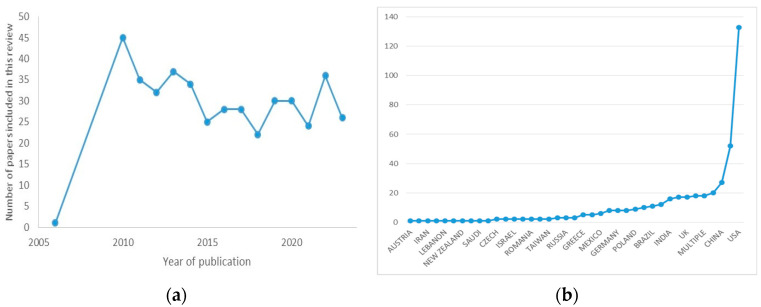
Graphical depiction of studies used for the literature review: (**a**) spread of articles used by date of publication; (**b**) spread of articles used in this review by location of research.

In our review, a total of 583 papers were identified, which became 579 after duplicate removal. Articles were excluded for the following reasons: (1) not relevant to the research topic (73 articles), (2) a lack of clear method/results details (11 articles), (3) language (3 articles), (4) retraction (2 articles), and (5) unavailable full text/abstract (65 articles). This study consisted of 27 case reports, 3 editorials, 1 drug guideline, 321 original research papers, and 81 reviews. All studies included in the analysis had at least one or more outcome measures available for one or more of the patient groups analyzed. A total of 425 studies were considered. A bibliography of the articles reviewed is shown in Figure 3.

The vast literature has shown the clinically effective IOP-lowering effects of bimatoprost. This drug has been extensively used in clinics and in research studies, thus it is vastly reported on in the literature on glaucoma and ocular hypertension. A large-scale UK-based study revealed that the bimatoprost–timolol fixed combination was only second to latanoprost–timolol prescriptions among 2015 patients [25]. Rahman et al. also reported that bimatoprost was the second most common drug that a sample of 1006 patients persisted with [26]. Heo et al. carried out a retrospective study into patients’ persistence with prostaglandin analogues and reported that bimatoprost was the least option that persisted compared to latanoprost and travoprost [27]. Bimatoprost is a drug of choice for non-respondent patients previously on other antiglaucoma medications [28,29]. Another study suggested that this may not be the case, as 83 non-responders in a clinical trial did not show an added statistical reduction in IOP with bimatoprost [30]. Crichton et al. reported an average, additional reduction of 10–15% after switching from other antiglaucoma medications to bimatoprost [31]. Furthermore, a large-scale pharmaceutical-based review suggested that there was better compliance to bimatoprost, 0.01% as compared to its 0.03% variant [32]. The bimatoprost–dorzolamide regimen has also been determined to improve retrobulbar blood flow, thereby inhibiting glaucomatous ocular morbidity [33]. Bimatoprost also retains its efficacy even after up to three years of use with minimal tolerance reports [34].

The local drops of bimatoprost are available in different concentrations and formulations. Bimatoprost is usually dispensed as an aqueous mixture with or without preservatives [35] and is also available as an ocular insert device. Its effectiveness may however be affected by its instability in aqueous solutions and isomerism [36]. The average washout period for prostaglandin analogues has been researched to be 4.56 weeks (±1.25) after the last application [37]. Lim et al. suggested that washout may take up to 6 weeks [38]. Bimatoprost has been found to be safe in breastfeeding mothers as it hardly gets into the bloodstream [39].

## 4. Selected Comparison Studies on Bimatoprost

### 4.1. Bimatoprost vs. Bimatoprost

Bimatoprost has been shown to be potent as a monotherapy and as a combination therapy [40,41]. As far as population-based efficacy reports, Bimatoprost has shown good efficacy among both primary open-angle glaucoma (POAG) and normal-tension glaucoma (NTG) patients in several Chinese and Japanese studies, respectively [42,43].

Konstas et al. examined IOP in 60 eyes being managed by bimatoprost and reported a drop from 29 mmHg to 20.8 mm Hg (*p* < 0.001) and combining bimatoprost with timolol resulted in a statistically significant average IOP reduction of 10.2 mmHg in the morning (*p* = 0.006) [44]. The presence or absence of preservatives such as benzalkonium chloride does not affect its efficacy [45,46]. Bimatoprost concentration affects its bioavailability in the aqueous humor with the 0.03% preparation, showing a statistically higher concentration in the aqueous after 30 min (11.5 ± 2.1 ng/mL versus 37.8 ± 28.8 ng/mL) [47,48]. This has been reported to enhance long-term outcomes [49]. Preservative-free bimatoprost 0.01% is reported to be less irritating [50], and non-cytotoxic as compared to the 0.03% variant that was reported to be cytotoxic and shown to reduce corneal endothelial cell viability after long-term use [51]. Bimatoprost 0.01% was found to reduce adverse ocular surface effects [52] while also maintaining efficacious IOP control than 0.03% and 0.0125% formulations of bimatoprost, respectively [53,54], with a drop of −4.9 ± 3.5 (*p* < 0.001). Patients who switched from 0.01% to 0.03% bimatoprost experienced a significant further reduction in IOP but with increased numbers of adverse effects, especially hyperemia [55]. Bimatoprost 0.01% monotherapy increased ocular perfusion pressure (OPP) in twenty hours of observing POAG patients [56] and extended treatment persistence in the chronic management of glaucoma [57].

### 4.2. Bimatoprost vs. Beta Blockers and α2 Agonists 

Prostaglandins became a first-line therapy for glaucoma management [58], partly due to their relatively easier once-daily dosing [59]. Natt et al. also reported similar superior IOP-lowering effects of bimatoprost when compared to brimonidine as shown in bimatoprost IOP reduction values of 8.9 ± 1.598 mmHg compared to 6.55 ± 1.26 mmHg among subjects that received Brimonidine [60]. Artunay et al. reported that brimonidine 0.2% was inferior to bimatoprost 0.003% for the prevention of post-YAG laser capsulotomy spikes in intraocular pressure [61].

### 4.3. Bimatoprost vs. Prostaglandin Analogues

Bimatoprost yields more IOP reduction than latanoprost in the preservative-free formulation [62]. Both aforementioned prostaglandin analogues (PGAs) have been reported to show greater first-line ocular hypotensive effects than timolol fixed combination agent adepts of PGAs; latanoprost had been suggested to pose good tolerability amongst study participants [63]. Novel preservative-free tafluprost 0.0015% has shown efficient IOP control with, of course, more improved tolerability than bimatoprost monotherapy [64,65,66]. 

As with other prostaglandin analogues, bimatoprost shows a stable reduction in IOP when compared with baseline values at 6-month and 5-year follow-ups [67]. The lower tonicity of 0.01% bimatoprost formulations has led to better treatment continuation outcomes among patients, superior to those of other PGA-based/first-line anti-OHT agents [68,69]. Tang et al. reported that bimatoprost 0.03% appeared more effective when used in long-term care as compared to other prostaglandin analogues [70], while Turan-Vural et al. suggested that Ketrolac enhanced the anti-glaucoma abilities of bimatoprost [71]. Nepafenac has also been shown to potentiate the effects of three major prostaglandin analogues: latanoprost, travoprost, and bimatoprost [72]. A clinical trial suggested that bimatoprost possessed the best efficacy of prostaglandin analogues during the initial phases of use [73] and among patients switched to bimatoprost from other antiglaucoma therapies [74]. The presence of nucleotide polymorphisms of the prostaglandin F-receptor (rs11578155 and rs6672484) has been described to be negatively correlated with prostaglandin analogues, including bimatoprost [75].

Bimatoprost is reported to be the most expensive antiglaucoma eye drop preparation available to most patients [76,77]. It has the second widest coverage under Medicare both in branded and generic forms [78]. Studies comparing the tolerability and suitability of bimatoprost and travoprost in patients revealed a statistically significant reduction in diurnal IOP as compared to standard latanoprost, i.e., 2.1 mmHg additional reduction in IOP with bimatoprost compared to 1.4 mmHg with travoprost; another study reported a 9.9 mmHg from baseline values for latanoprost as against 15.88 nnHg for bimatoprost [79,80,81]. The drug showed better quality of life indices as compared to latanoprost, travoprost, and tafluprost [82]. Among prostaglandin analogues (PGAs), bimatoprost has been reported to exert better diurnal IOP control in glaucoma monotherapy than latanoprost single therapy and is poised to exert greater intraocular pressure control, with Mishra et al. reporting the highest reduction with a 35.9 ± 3.5% reduction in IOP compared to 29.9 ± 3.5%, 30.8 ± 2.6% and 26.6 ± 2.3 for latanoprost, travoprost, and timolol respectively [83,84]. Kook et al. also reported as part of the APPEAL study that switching participants from other therapies to bimatoprost 0.01% single therapy resulted in a statistical decrease in IOP [85]. More so, BTFC was preferred to travoprost by patients [86,87].

Bimatoprost could be linked with a lower comparative number of non-respondents in NTG [88]. Findings by Sato et al.; Sawada et al.; and Germano et al. suggested that transitioning from latanoprost to bimatoprost yielded better therapeutic effects; furthermore, latanoprost was noted to be the least tolerated among all prostaglandin analogues [89,90,91,92]. Bimatoprost, travoprost, and latanoprost have been reported to portend equivalent ocular surface tolerability with medium-term use [93]; latanoprost and travoprost yielded more visible ocular hyperemia than bimatoprost in human and murine studies, respectively [94,95]. The type of prostaglandin analogue preservative had no significant effect on ocular side effects [96]. Toxic effects of topical prostaglandin analogues bimatoprost, travoprost, and latanoprost on rabbit eyes were more dependent on the concentration of BAK preservative than agent selection [97]. Bimatoprost had a stronger association with the deepening of the upper eyelid sulcus (DUES) among Japanese patients, following a switch from latanoprost [98,99]. Other authors have, however, suggested that switching from fixed prostaglandin therapy to combination therapies which include beta blockers can help reduce hyperemia [100]. Bimatoprost has been subjectively linked to increased hyperemia and more side effects in human patient studies [101,102,103]. A keratograph may suggest better objective automated grading of ocular hyperemia [104].

Topical PGAs tend to have high-cost implications when initiated with adjunctive therapy [105,106]. For many years, latanoprost has been the most common prostaglandin analogue used as a topical therapy for glaucoma, accounting for about 65% of prostaglandin prescriptions [107]. Latanoprost had been associated with more persistent periods of use, probably due to lower cost implications than bimatoprost and travoprost [108,109]. Bimatoprost has, however, been linked with higher first-year expenditure than both travoprost and latanoprost [110]. Slower rates of glaucoma progression for patients on bimatoprost have, however, been theorized to increase long-term cost savings in their favor [111]. Islam and Spyry also opined that bimatoprost was the most cost-effective prostaglandin analogue in the Canadian community [112]. Eyes with thinner cornea centers may be more responsive to the antihypertensive effects of topical bimatoprost [113]. This may have to do with its comparatively reduced corneal tissue permeability [114]. Bimatoprost 0.01% demonstrates good treatment outcomes in clinical studies [41,115].

Tafluprost remained inferior to bimatoprost as an IOP-regulating agent [116,117] except when optimized with benzalkonium chloride [118]. Bimatoprost formulation has been reported to maintain optimal stability under various thermal conditions, unlike travoprost and latanoprost [119]. Bimatoprost 0.01% also compares to travoprost 0.04% in controlling late-day IOP levels [120]; Bimatoprost 0.03% has on the other hand, been suggested to control IOP better than travoprost at 12 weeks following onset of use [121].

Adverse periocular manifestations are common with topical use of PGAs [122]. Kucukevcilioglu et al. compared periorbitopathy with latanoprost, bimatoprost, and travoprost, concluding that these were most severe and frequent in the bimatoprost-treated group [123,124]. Long-term use of bimatoprost may affect periorbital cosmesis largely by significant inhibition of adipogenesis by bimatoprost acid in vitro [125,126]; this was suggested in a case report as well [127]. Similarly, switching away from bimatoprost therapy may lead to an improvement in DUES [128].

### 4.4. Bimatoprost vs. Multi-Class IOP Lowering Medications 

While timolol has often been touted as the most cost-effective anti-glaucoma drug, bimatoprost was reported as the most effective alternate option in situations where beta blockers are contraindicated [129]. Other studies recommend prostaglandin analogues as first-line treatment for glaucoma [130,131].

A review of practice patterns by eyecare practitioners in the United States outlined bimatoprost among commonly used first-line agents [132]. A survey of 150 Mexican Ophthalmologists revealed bimatoprost was the most preferred prostaglandin analogue prescribed, with 62% of respondents preferring the drug [133]. Another study reported that bimatoprost comparatively increased OPP when compared to Timolol [134]. Prostaglandin therapies are associated with lesser diurnal fluctuations in intraocular pressure control than other mono-therapeutics [135,136]. It is therefore not unexpected that a bimatoprost-timolol fixed combination is one of the most tolerated and satisfactory pressure-lowering regimens in POAG [137]. The same holds true in patients with chronic angle closure glaucoma [138]. In fact, the IOP-lowering effects of bimatoprost have been clocked to persist longest amongst the PGAs at 48 h after a single dose [139]. Fixed combination bimatoprost/timolol was reported to reduce IOP more optimally than dorzolamide/brimonidine/timolol combination drops [140]. Brief et al. also reported that BTFC is associated with good tolerability and adherence among the majority of participants in glaucoma/ocular hypertension treatment studies [141]. It has been suggested that morning use of bimatoprost with dorzolamide hydrochloride as an adjunctive agent also reduced diurnal IOP variation [142]. Reports from the APPEAL Taiwan Study reaffirmed the clinical usability of bimatoprost 0.01% [143], however, prostaglandin analogs generally have been associated with thickening of the macula, but Selen et al. have suggested that there are no significant inter-drug differences [144]. 

### 4.5. Bimatoprost vs. Fixed Combination Therapies

Common anti-glaucoma combination drugs usually consist of a prostaglandin analogue and a beta-blocker [145,146]. They are especially important in patients who do not have a satisfactory IOP reduction in IOP with monotherapy [147,148]. Prostaglandin-timolol fixed combinations (PTFCs) have generally demonstrated improved treatment efficacy over non-fixed formulations [149,150,151,152,153], reduced individual PGA-induced hyperemia [154,155], cost implications [156], potentially regularized dosing regimen [157,158], and improved long-term adherence to treatment [159,160]. In Belfort et al.’s study BTFC patients reported lower levels of hyperemia with prostaglandin fixed combination drops [161]. Bourne et al. have differed on this, stating that switching patients from a BTFC to a tafluprost-timolol combination mitigated hyperemia signs [162].

Prostaglandin/timolol unfixed combinations were tagged as more effective than PTFCs in a pilot study [163,164]. Notably, the therapeutic effects of PTFCs varied with the choice of PGA component in formulations [165]. Bimatoprost single therapy, however, was equally comparable to latanoprost–timolol fixed combination in reducing IOP strength according to Maruyama et al.’s study [166]. The diurnal IOP-control effects of bimatoprost monotherapy were reportedly equal to the latanoprost/timolol fixed combination [167,168,169,170]. Bimatoprost–timolol fixed combination drops were reported by Lee et al. to further reduce IOP in patients who had been switched from separate latanoprost and timolol drops [171]. 

Ganfort, a preparation of bimatoprost/timolol was reported to better decrease IOP in a cohort of patients with neovascular glaucoma [172,173], as well as treatment-naive and NTG patients [174,175]. Bimatoprost/timolol fixed combination reportedly exerted greater IOP reduction than travoprost/timolol and latanoprost/timolol in open-angle glaucoma patients [176,177]. Hartleben et al., however, reported slightly different findings, stating that triple agent combinations containing bimatoprost performed better than bimatoprost–timolol fixed combinations [178]. A triple-fixed combination of anti-glaucoma also performed better than a double-fixed combination in the study by Menon and Goodkin, albeit in poorly controlled patients [179]. Interestingly, Sussana et al. reported that a once-daily administration of the triple-fixed therapy bimatoprost–timolol-brimonidine performed better than a bi-daily administration of the same drug [180].

Travoprost/timolol and latanoprost/timolol were previously assumed to achieve better IOP control effects than several other common agents in fixed combinations with Timolol [181]. Nevertheless, bimatoprost/timolol formulations have demonstrated good efficacy, even without benzalkonium chloride (BAK) preservatives [182,183]. A study reports improved treatment outcomes upon transition from bimatoprost 0.03%/timolol 0.5% to travoprost 0.004%/timolol 0.5% in resistant OHT cases [184]. Bimatoprost monotherapy demonstrated equivalence to latanoprost/brinzolamide as valuable transition options following prior latanoprost monotherapy [185]. A significant reduction in hyperemia was found after switching from monotherapy with prostaglandins or prostamide to a fixed combination of prostamide and a β-blocker [186]. Conversely, a proportion of patients who transitioned from a bimatoprost/timolol to a travoprost/timolol fixed combination reportedly gained improvements in IOP and tolerability with their new therapy [187]. Once-daily BTFC has been found to be more effective in mean IOP control than the dorzolamide/timolol combination used twice daily [188] or latanoprost/timolol [189], with no differences found in the safety profiles of both agents [190]. 

Pfennigsdorf et al. reported that BTFC lowered mean IOP from baseline by 5.4 mmHg over the 12-week duration of the studies (*p* < 0.0001) [191]. BTFC, latanoprost/timolol, and travoprost/timolol fixed combinations reportedly yield equivalent degrees of tolerability for patients requiring adjunctive topical agents [192]. When side effects were measured objectively using factors such as hyperemia, no significant difference was found between PGAs [193]. A prospective study of the effects of bimatoprost as compared to dorzolamide–timolol showed comparable IOP-lowering effects for both drugs [194]. Inference from the United Kingdom general practitioner research database suggested that lower-cost travoprost-centric therapies were linked with longer treatment resistance than other PGA-centric therapies [195]. Xu et al. also suggested in a study out of China that Bimatoprost was the most cost-effective among other anti-glaucoma drugs [196]. Moreover, across the continent in Russia, Kosakyan et al. also recommended bimatoprost for its comparatively minimal adverse effects and hypotensive properties [197].

The evaluation of hyperemia caused by ocular medications can be very subjective; to this end, Sumi et al. developed an automated hyperemia analysis computer-based software [198]. Clinical decisions on which therapy to recommend are achieved by collating results across multiple studies. Kymes et al. were able to demonstrate an artificial intelligence tool that correctly analyzed multiple publications, analyzing their results and IOP endpoints, before they eventually concluded that bimatoprost comparatively achieved a better IOP drop when tested with other prostaglandin analogues [199], notwhitstanding possible side effects of prostaglandins generally [200]. A comparative analysis of the IOP-lowering effect of bimatoprost is depicted in Table 1 below.

## 5. Complications of Bimatoprost

As with any chemotherapeutic agent, there are bound to be complications with the use of bimatoprost. Systemically, prostaglandins have been reported to cause contraction of the uterine muscles and eventual spontaneous abortion. However, there are not enough data to confirm if this same complication is present in topical prostaglandin analogs [207]. Tanito et al. recommended a grading system for prostaglandin-associated orbitopathy [PAP] [208]. They found that bimatoprost was associated with higher PAP grades as compared to tafluprost and omidenepag. This was echoed by Hikage et al., who reported that omidenepag may not cause sunken eyeballs [209]. PAP has been reported to be positively associated with bimatoprost and age, while a high BMI gave a negative correlation [210]. Sarnoff and Gotkin suggested adjusting the mnemonic from PAP to PAPS, to accommodate the words ‘periorbital syndrome’ to capture the beneficial properties of bimatoprost [211]. Ida et al. have recently demonstrated in vitro that bimatoprost acid inhibited adipogenesis, and its action is also potentiated by the addition of an EP2 agonist such as omidenepag [212]. Ocular hyperemia after topical instillation of bimatoprost is also a recurring theme in studies sampled [213,214]. Some of these complications are listed in Table 2.

Liang et al. suggested that human corneal endothelium (HCE) cytotoxicity as reported amongst prostaglandin analogues was at least partly caused by preservatives [261]. Their research demonstrated that preservative-free tafluprost showed the least HCE cytotoxicity as compared to other prostaglandin analogues containing different concentrations of benzalkonium chloride. Benzalkonium chloride itself has been implicated to exacerbate dry eye symptoms in study mice [262,263] and also delay wound healing [264]. Nakakura et al. [265], however suggested that sunken eyelids secondary to bimatoprost use can be reversed by switching patients to latanoprost. Altieri and Ferrari also disputed that bimatoprost use resulted in eyelid position changes [266]. Radcliffe has also suggested that the addition of timolol to bimatoprost to be dispensed as a single fixed combination drop greatly reduces the risk of hyperemia as a side effect [267]. Other studies suggested that Rho-kinase inhibitors may inhibit the effect of bimatoprost on orbital tissue [268,269]. Specifically, preservative-free bimatoprost–timolol may be the most tolerated anti-glaucoma fixed combination drug [270,271,272].

Ocular adverse events included pruritus, periocular hyperpigmentation, hypertrichosis, and irritation [273,274]; these were deemed easily reversible upon discontinuation of bimatoprost ophthalmic solution among those who switched medications [275]. On average, PTFCs induce more conjunctival infiltration [276]. Ocular adnexal, specifically conjunctival and palpebral infiltration of macrophages were associated with long-term use of preservative-free bimatoprost. These effects were hypothesized to be owing to residual excipients within the solution [277]. When added to bimatoprost, benzalkonium chloride, polyoxyethylene hardening castor oil 40 (HCO-40), and polysorbate 80 (P-80) have demonstrated toxicity to corneal and conjunctival cells in vitro [278]. Alternate analysis of conjunctiva-associated lymphoid tissue changes in rabbit models found common inflammatory infiltration changes for all PGAs preserved with BAK [279,280]. In contrast, latanoprost and tafluprost were linked with a reduction in the cytotoxicity of BAK on human conjunctival cell cultures [281]. BAK concentrations in bimatoprost also had cytotoxic effects on in vivo models of mature human adipocytes and preadipocytes [282]. Bimatoprost-induced DUES was ameliorated following its discontinuation [283].

Upregulation of matrix metalloproteinase expression via human non-pigmented ciliary epithelial cells bodes potential aggravation of prior low-grade inflammatory processes within the eye following consistent and prolonged topical instillation of bimatoprost, as with several other PGAs [284]. There were sparse case reports of bimatoprost use associated with choroidal detachment in the eyes of elderly persons; previous intraocular surgery seemed to be a prevailing risk factor in affected eyes [285].

Much like most antiglaucoma drop formulations containing a non-selective beta-blocker, BTFC was linked with an alteration of normal pulmonary physiology in a case report [286]. Situations in which topical drops alone cannot reduce IOP to acceptable levels may necessitate glaucoma surgery. Bimatoprost use pre-surgery may result in recurrent IOP elevation up to two years after trabeculotomy [287].

**Figure 4:**
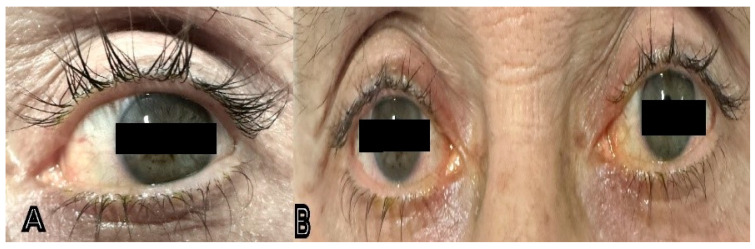
External ocular complications of bimatoprost use: (**A**) eyelash darkening, DUES, and growth in a 70-year-old male patient using bimatoprost for POAG; (**B**) 63-year-old glaucomatous female patient with DUES, mild conjunctival hyperemia, ocular dryness, iris darkening, eyelash growth, and periorbital pigmentation.

Psychometric analyses via quality-of-life questionnaires were used to assess patients’ outlook toward the side effects of bimatoprost among other anti-glaucoma drugs. Results of this study concluded that respondents were more concerned by the side effects of bimatoprost over other anti-glaucoma drugs tested [288]. Some complications of bimatoprost are shown in Figure 4 below.

## 6. Bimatoprost in Other Fields

Bimatoprost indications have grown beyond ophthalmology and dermatology, with ongoing research exploring its applications in glaucoma surgery, neuroprotection, and even cosmetic procedures [289,290,291]. Care, however, must be taken to be wary of the effects of eye cosmetics on the eye in general [292]. Gregorio et al. reported on an interesting case of unpredicted resolution of floppy iris syndrome and ectropion in a geriatric patient after unrelated management for glaucoma using bimatoprost [293]. 

In dermatology, bimatoprost has shown promising results in the promotion of hair growth, particularly in the treatment of alopecia/hypotrichosis [294,295,296,297,298], leukoderma [299,300], and for general eyelash enhancement [301,302]. Its use in this respect was fully approved by the Food and Drug Authority in 2008 [303]. Alopecia areata is a condition presenting with variable hair loss [304,305] and can be a side effect of anti-cancer chemotherapy [306,307]. A common treatment for alopecia areata is the steroid Clobetasol propionate cream [308] and Pentoxifylline [309]. Bimatoprost induced eyelash growth over one year in a portion of patients who had alopecia areata [310,311,312]. It was also shown to be effective in steroid-resistant cases [313]. Anbar et al. reported that three different prostaglandin analogues (including bimatoprost) were capable of producing skin pigmentation in murine models [314]. Its mechanism of action, stimulating the hair cycle and prolonging the growth phase, has led to the development of bimatoprost-containing solutions for enhancing eyelash growth [315]. This has been well-received by patients seeking longer, fuller eyelashes [316]. Improvement of lash properties may be dependent on the location of the hairs on the face [317]. There are, however, ethical issues to be resolved in this percept. Banazek [318] warned that the complications of bimatoprost use far outweigh the potential cosmetic applications.

Subedi et al. formulated an enhanced bimatoprost preparation named BIM-TF#5 and it was found to be five times more potent than bimatoprost-in-ethanol in dermal drug penetration [289]. Lasers are indicated for enhancing the uptake of drugs into skin tissue [319]. Waibel et al. compared three treatment modalities for managing skin hypopigmentation, namely fractional lasers, laser-mediated bimatoprost delivery, and a dermal melanocyte harvesting technique [320]. There was a statistically significant improvement in hypopigmentation in laser-mediated bimatoprost delivery as compared to the other two techniques. Thus, bimatoprost has been reported for its ability to induce melanogenesis and hyperpigmentation. Nguyen et al. [321] suggested that micro-needling hypopigmented burn scars with bimatoprost is a viable way to induce darker tones on skin tissue. Other authors reported that a combination of fractional ablation and bimatoprost therapy resulted in the repigmentation of hypopigmented skin tissue [322,323]. Regis et al. also reported a case series of two patients presenting with hypopigmented scars on their lower legs following treatment laser hair removal procedure. Both patients showed complete repigmentation after treatment with fractional resurfacing and topical bimatoprost therapy [324]. Following the pandemic, various novel medical complaints and conditions were presented and new therapies had to be formulated. Acute respiratory distress is a common symptom seen in COVID-19 patients and its management involves prolonged prone positioning resulting in facial lesions. Laser-mediated bimatoprost delivery with microneedling was shown to reverse pigmentation [325].

Another dermatological concern managed with bimatoprost is vitiligo [326,327,328]. Vitiligo may occur non-segmented or segmented. Using the Vitiligo Area Scoring Index (VASI), Silpa-Archer et al. were able to demonstrate that bimatoprost combined with NB-UVB therapy improved the scores in patients with non-segmented vitiligo, while Jha et al. demonstrated same using photographs [329,330]. This NB-UVB technique was also used in addition to fractional carbon dioxide laser to achieve repigmentation in non-facial vitiligo [331].

Idiopathic guttate hypomelanosis is a condition associated with pale patches on the extremities. Kreeshnan and Madan published a case report detailing how they used bimatoprost in combination with a 308 nm excimer light, reversing pigmentation loss [332].

## 7. Bimatoprost and Hair Growth

Research shows success with the off-label aesthetic use of bimatoprost with varying disorders [333,334], although study groups sampled have lacked adequate racial diversity [335]. PGAs are constituents in certain over-the-counter cosmetics for lash enhancement [336]. Bimatoprost is emerging as an important player in cosmetics, albeit sometimes illegally [337]. Faigen used a patient-reported outcomes (PROs) questionnaire to assess the experience of 278 adults treated with bimatoprost for eyelash extensions and reported that cutaneous application of bimatoprost 0.03% produced favorable safety and tolerability [338]. Dilute levels of bimatoprost applied cutaneously for hair growth were found in the blood of test mice [339]. Bimatoprost gel and 0.03% solution both ameliorated madarosis post-chemotherapy in study groups [340,341,342]. Rossi et al. further advised that bimatoprost only be used after chemotherapy and not before or during chemotherapy [343]. Bimatoprost is reported to produce quantifiable enhanced hair and lash growth within one to two months of the commencement of the regimen [344,345], although female pattern androgenetic alopecia failed to respond to an injection of bimatoprost 0.03% [346]. The hair-growth potentials of bimatoprost were considered for possible treatments of androgenic alopecia [347,348]. Theoretically, bimatoprost was suggested to stimulate scalp follicles, potentially remedying scalp alopecia [349]. Bimatoprost achieves this by stimulating hair in the telogen stage to the anagen stage [350]. Chronic forms of alopecia, specifically alopecia totalis and universalis, may not show eruption of new hairs, but existing hairs may be lengthened after bimatoprost treatment [351]. Lu et al. examined common cosmetic products for the presence of prostaglandin analogues using nuclear magnetic resonance and other techniques, discovering a trend of illegal addition of bimatoprost and latanoprost as ingredients in these items [352]. Johansson et al. and Lee et al. also confirmed the presence of illegal supplementation with bimatoprost inside eyelash extension preparations [353,354]. Bimatoprost has also been reported as a management option for androgenetic alopecia [355] and for eyebrow rejuvenation [356,357].

Bimatoprost is safe in children [358] as in adults and has been prominently implicated in the literature to cause elongation of facial hair and has been reported to be as potent as Minoxidil [359,360,361]. Using a baseline of three factors, namely length, fullness, and satisfaction with hair, Fagien et al. demonstrated that bimatoprost 0.03% applied topically outperformed control groups in a multicenter study [362]. Another scale used is the Global Eyelash Assessment scale, and with this scale, Kwon et al. reported that bimatoprost use resulted in the safe elongation of the eyelashes of participating Asian females [363]. Use of bimatoprost 0.03% gel over a six-month period resulting in desired cosmesis with the enhancement of the eyelashes [361,362]. When compared to a placebo, bimatoprost produced statistically significant elongation of eyelashes, establishing that the extension obtained with this drug did not occur due to chance [364,365,366]. On the bright side, bimatoprost does not cause iris pigmentation when used periocularly and not as an eyedrop [367]. Bimatoprost has also been reported to be safe and well-tolerated as a therapy for the loss of eyebrow hair [368]. To this end, Suchonwanit et al. showed that 0.03% bimatoprost improved eyebrow trichosis as compared to bimatoprost 0.01% [369].

Hereditary linked progressive loss of scalp hair can also benefit from bimatoprost therapy [370]. Hypotrichosis can also be secondary as in the case of its chemically induced variant. Bimatoprost 0.03% has again been reported to induce hair growth in chemically induced hypotrichosis [371,372]. Electron microscopy revealed that bimatoprost-treated hairs possessed thicker dermis [373]. Chanasumon et al. suggested that bimatoprost’s restorative mechanism involves stimulation of the PGF2α and PGE2 receptors that potentiate hair growth [374,375,376]. Wirta similarly confirmed the ability of bimatoprost to simulate hair growth in a pooled study [377]. However, care should be taken in this regard, as Wirta et al. pooled a study of six multicentered studies lasting at least three months and reported that significant adverse effects were associated with the management of hypotrichosis using bimatoprost. However, the microneedling technique administered with vitamin D3 combined with bimatoprost is reported to improve hair production as compared to treatment with bimatoprost or vitamin D3 alone [378]. Patients may potentially avoid/reduce unwanted eyelash-lengthening effects of bimatoprost by using an absorbent material to wipe off excess eye drops after installation [379]. Palmer et al. examined the effect of bimatoprost 0.01% on nail growth and found that changes were not significant after one month of treatment [380].

## 8. Evolution of Bimatoprost Therapies

Drug compliance and persistence is a major problem in patients on long-term medication for chronic conditions like glaucoma [381]. The evolution from traditional eye drops to sustained-release implants and timolol gel-forming solutions has enhanced patient compliance and convenience [382]. These formulations offer extended drug delivery, reducing the frequency of administration and minimizing side effects [383]. With these, bimatoprost’s global use has expanded significantly, with healthcare professionals recognizing its potential benefits.

Implantable drugs and sensors can come to the rescue for such patients [384]. In such patients, a single intracameral insert may solve this problem and remove the need for a daily regimen of drugs [385]. Intracameral delivery of drug formulations achieves the double aim of bypassing the selective layers of the cornea and also ensuring instant bioavailability of the administered agent [386]. Mathematical models predict that intracameral slow-release devices are capable of achieving between 26–30% IOP reductions over a 6-month period [387]. Bimatoprost is also available as a sustained-release injection and this has been reported to lower pressure and thereby reduce multi-drug therapies even in post-selective laser trabeculectomy patients [388]. Some researchers have suggested using these sustained-release devices as an additive therapy against using them as an alternative to topical instillation [389]. However, the dose response for bimatoprost sustained-release devices in lowering IOP was more consistent than that obtained with topical instillation. Lewis et al. have shown the good tolerance and IOP-lowering abilities of bimatoprost implants after 6 months [390]. A single implant is potentially capable of delivering consistent IOP-lowering medication and results for years [391] and even up to two years [392]. These implants are available in different doses and have been found to have dose-dependent IOP-lowering effects [393]. Examples of other devices include nVISTA [394], iDose, OTX-TP, Latanoprost free acid SR, and ENV515, which are in different stages of trials [395]. Xu et al., however, reported that 15 of 48 eyes fitted with a bimatoprost sustained-release device suffered a device failure [396]. An intracameral implant was recently studied by Sirinek and Lin [397] and they noted that corneal endothelial cell loss is a possible complication of these devices.

Satyanarayana et al. reported on a novel bimatoprost delivery formulation using solid nanoparticles [398]. Studies on canine eyes revealed that bimatoprost SR significantly maintained lower IOP than topical administration [399]. Mean venous pressure has also been shown to reduce after bimatoprost use significantly [400]. It, however, remains to see how this novel delivery will be tolerated in the human eye in vivo, and what will be the best delivery method if well tolerated. Yadav et al. reported on a novel nano vesicular sustained release device designed to carry bimatoprost [401]. In vivo and in vitro insertion of the device was reported to be non-problematic without any resulting inflammatory signs [402]. Conjunctival fornix sustained-delivery inserts containing bimatoprost were tested in vivo using murine models and had good IOP reduction indices [403]. Topical bimatoprost inserts translated well to patient groups: they were associated with good long-term IOP control and insert retention in a phase II clinical trial [404].

Furthermore, these sustained-release devices can still be used safely in the eye that has undergone selective laser trabeculoplasty [405]. The ARTEMIS study evaluated the biodegradation rates of bimatoprost implants in 372 eyes, concluding that while there was statistical variability between sampled eyes, the majority of eyes showed significant biodegradation of implants after one year [406].

## 9. Novel Delivery Technologies for Bimatoprost

Promising future perspectives for bimatoprost include the exploration of novel delivery systems, such as contact lenses [407] to enhance drug efficacy and patient comfort. Through various formulations, advancements, and global use, bimatoprost has shown immense potential for addressing a wide range of therapeutic needs.

Sustained release delivery of drugs via contact lens is an emerging field due to the popularity of contact lens wear and its non-invasive status [408]. This, however, has implications for the dose delivery levels in patients wearing contact lenses. For example, Lotrafilcon-A-based contact lenses have been reported to show an uptake of up to 86% for bimatoprost contained in a 60 μL drop within one hour, leading researchers to suggest that wearers remove such contact lenses before the use of bimatoprost [409]. Graphene oxide fortification of contact lens polymers has been shown to enhance bimatoprost affinity to contact lenses and amplify drug delivery, albeit in murine models [410]. Vitamin E impregnation at a rate of 0.2 g per gram of hydrogel is reported to prolong the bimatoprost adherence and delivery dynamics over 10-fold [411]. Contact lenses loaded with gold nanoparticles have also been shown to increase drug retention and give better distribution of bimatoprost over time [412]. A novel biodegradable intracameral delivery technology passed phase 1 FDA approval [413]. Allergan’s single-use implant, “Durysta”, could potentially elongate treatment effects in glaucoma patients [413].

Nanosponge technology is another new medical innovation in eye care. Lambert et al. reported that bimatoprost-loaded nanosponges were able to lower IOP by up to 30% in mice that had artificially induced ocular hypertension [414].

## 10. Future Perspective for Bimatoprost

Ongoing research and future perspectives continue to shape its role in medicine, promising further advancements and improved patient outcomes. The aim of our review is to provide a brief description, overview, and studies in the current literature regarding the versatile clinical use of bimatoprost over the past 25 years. This can help clinicians determine the most suitable individualized therapy to meet the needs of each patient.

Bimatoprost has been described in the literature as the best agent for ocular hypertension and glaucoma management [415]. Efforts made in developing even more stable novel formulations are bound to be worthwhile. Because of its popularity and acceptability, bimatoprost may be easily abused seeing as it is a long-term, occasionally over-the-counter medication [416].

Despite the well-documented adverse PAP effects of bimatoprost, this very phenomenon suggests possible therapeutic use in resolving orbital proptosis in patients with Grave’s orbitopathy (GO) [417]. Draman et al. reported a reduction, though insignificant, in exophthalmometry results in patients suffering GO after a three-month regimen of bimatoprost [418]. Melovatskiy et al. have also theorized a similar application of bimatoprost to mitigate thyroid orbitopathy [419]. Eiger-Moscovich et al. conducted a blind study to assess the effects of bimatoprost on ocular tissue in patients with inactive thyroid orbitopathy’ they opined that bimatoprost reduced periorbital fat tissue expansion on visual observation [420]. Practically, Higashiyama and Ohji reported that a year-long bimatoprost therapy did not significantly reduce the extraocular fat volume or extraocular muscle volume [421].

The very effects of prostaglandins as documented In decreasing the corneal endothelial cell count suggest that caution be taken in the measurement and prescription of these classes of drugs to chronic glaucoma patients [422]. Proper diligence must be taken by periodically standardizing intraocular pressure measurements using pachymetry. Evolving technologies such as the automated visual field [423], scanning laser polarimetry [424], and dynamic contour tonometry [425,426] show promise in helping better manage glaucoma.

Adapting automated means of analysis into clinical trials centered on varying formulations of bimatoprost may more reliably facilitate the determination of adverse drug events with better objectivity [427].

Bastia et al. [428] examined retinal cell physiology restorative properties of different preparations of bimatoprost. They induced retinal cell disruption by administration of endothelin-1 and observed significant changes in electroretinogram patterns. Studies based on special formulations of bimatoprost treatment (NCX 470) subsequently returned values to baseline after six weeks. NCX 470 is a nitric oxide-donating prostaglandin analog. This lends credence to the ability of bimatoprost to offer neuroprotection [429]. NCX 470 is also more effective than latanoprost [430] and equimolar bimatoprost for IOP reduction in canine models [431,432]. Studies based on a special 0.01% preparation of bimatoprost, named JV-GL1, with a prostaglandin E2 receptor, have also shown tremendous promise by producing better IOP-lowering results than bimatoprost 0.03% [433].

## 11. Conclusions

Prostaglandin analogues will continue to be the mainstay of glaucoma management going into the next few decades. Bimatoprost ranks as arguably the most potent antiglaucoma and anti-OHT agent available on the open market. However, the data available do not give a definitive picture of its relative standing in comparison to other antiglaucoma drugs. However, more work still needs to be done as regards its adverse effects, especially hyperemia. Hirsutism, lash elongation, and skin pigmentation are amongst some of its side effects, but interestingly, these can also be clinically desired in other patients. Further research will be required in how to standardize bimatoprost preparations for use in extraocular conditions.

## Figures and Tables

**Table 1:** Studies regarding the comparative IOP-lowering effects of bimatoprost.

Study and Year	Design	Study Population	Main Outcomes with *p* Values
Holstrom et al., 2005 [201]	Meta-analysis	9295 (42 studies)	30.3% reduction in IOP from baseline with bimatoprost compared to 28.7% with travoprost and 26.7% with latanoprost (*p* = 0.001)
Denis et al., 2007 [202]	Meta-analysis	1318 (6 studies)	bimatoprost produced the largest reduction in IOP (−1.04 mmHg, 95% CI: −2.11; 0.04; *p* = 0.06) compared to bimatoprost (−0.98 mmHg, 95% CI: −2.08; 0.13; *p* = 0.08) and a reference latanoprost treated population
Noecker et al., 2003 [203]	Clinical Trial	31	bimatoprost produced a mean IOP reduction of 8.4 mmHg (33.7%) compared to 7.9 mmHg (30%) with travoprost (*p* = 0.671)
Noecker et al., 2003 [204]	Clinical Trial	269	From baselines before treatments of 24.0 ± 0.27 mm Hg (bimatoprost) and 23.3 ± 0.27 mm Hg (latanoprost), IOP reduced statistically more in the bimatoprost group as compared to the latanoprost group (16.5–17.4 mm Hg; 17.6–18.9 mm Hg, *p* ≤ 0.008)
Parrish et al., 2003 [205]	Clinical Trial	393	Three separate groups of participants achieved IOP lowering of 8.6 ± 0.3 mmHg, 8.7 ± 0.3 mmHg, and 8.0 ± 0.3 mmHg when treated with latanoprost, bimatoprost or travporost respectively (*p* = 0.128 for difference among groups).
Cantor et al., 2004 [206]	Clinical Trial	26	IOP reduced by 7.4–8.8 mmHg with bimatoprost as compared to 4.6–7.2 mmHg with travoprost (*p* = 0.057)

**Table 2:** Summary of complications related to bimatoprost.

Summary of Findings	Formulation	Location of Complication	Authors
Bimatoprost use was implicated in the sudden expression of non-vellus hairs in a female patient	Lumigan 0.03%, (Allergan, Dublin, ON, Canada)	Face	Kaliaperumal et al. [215]
The bimatoprost regimen was associated with a reduction in IPD	Lumigan 0.03%, (Senju Pharmaceutical Co., Osaka, Japan)	Interpupillary distance	Sano et al. [216]
Bimatoprost is implicated in eyelash trichomegaly	Bimatoprost *	Eyelash	Hutchinson et al. [217]
The doctor noticed audible clicking of eyelashes secondary to eyelash elongation	Bimatoprost *	Eyelash	Skorin and Dailey [218]
Bimatoprost produced more upper eyelid changes as compared to latanoprost	Bimatoprost 0.03% *	Eyelid	Karslioglu et al. [219]
Hypopigmentation progressing to Lentigo Maligna Melanoma	Bimatoprost 0.03% *	Eyelid tissue	Deveau et al. [220]
Bimatoprost use resulted in eyelid tightening and shortening in the mouse model	Bimatoprost 0.03% *	Eyelid tissue	Kent and Custer [221]
Bilateral upper lid retraction was observed with bimatoprost use	Bimatoprost 0.03% *	Eyelids (upper)	Noma and Kakizaki [222]
DUES was the most common complication seen in an Asian study	Bimatoprost *	Orbit	Kim et al. [223]
Topical and retrobulbar injection of bimatoprost resulted in a drop in adipocyte cell count	Lumigan 0.03%, Allergan	Orbital fat	Jbara et al. [224]
Orbital fat reduced as evidenced by adnexal photographs	Bimatoprost *	Orbital fat	Rabinowitz et al. [225]
MRI and computer image algorithms respectively showed reduced orbital fat after bimatoprost use	bimatoprost (Allergan, Inc.),	Orbital fat	Higoshima et al. [226], and Eftekhari et al. [227]
Prostaglandins inhibit adipogenesis resulting in DUES	Bimaopost and Bimatoprost acid (Cayman Chemical Co., Ann Arbor, MI, USA)	Orbital tissue	Taketani et al. [228] and Ida et al. [229]
Bimatoprost implicated in peri-orbitopathy	Bimatoprost *	Periorbital	Tan and Berke [230]
Atrophy of the eyelid levator muscle was observed after the use of bimatoprost monocularly	Lumigan 0.03%, Allergan	Periorbital muscle	Wang et al. [231]
Bimatoprost (Latisse) induced skin discoloration	Bimatoprost solution, 0.3% (Latisse; Allergan Inc., Dublin, ON, Canada)	Periocular discoloration	Priluck and Fu [232]
Adverse effects include conjunctival hyperemia, burning sensation, and growth of eyelashes	Bimatoprost 0.03% *, Bimatoprost 0.01% *	External eye	Lewis et al. [233] and Park et al. [234]
Hyperemia and eyelash elongation were the most common side effects reported in a large Asian study	Lumigan 0.03%, Allergan	external eye	Sun et al. [235]
Bimatoprost use resulted in orbital fat prolapse and iris pigmentation	Lumigan 0.03%, Allergan	Eyeball tissue and iris	Aydin et al. [236], Jayaprakasam and Ghazi-Nouri [237], and Park et al. [238]
The tear profile of patients on bimatoprost revealed elevated cytokines mediating allergenic pathway	Bimaotoprost 0.01% *	Tears	Reddy et al. [239]
Bimatoprost scored highest on the pain scale in cataract surgery patients	Bimaotoprost *	Anterior eye	Ulas et al. [240]
A slight but significant increase in thickness observed	Bimatoprost 0.03% *	Central corneal thickness	Bafa et al. [241]
A slight but significant reduction in thickness observed	Bimatoprost 0.03% *	Central corneal thickness	Zhong et al. [242] and Eraslan and Celikay [243]
A systematic review showed that prostaglandin analogs result in thinning of the CCT	Bimatoprost *	Central corneal thickness (CCT)	Kim and Yim [244]
Bimatoprost was associated with conjunctival hyperemia	Lumigan PF 0.03%	Conjunctiva/cornea	Wu et al. [245]
PGF2α including bimatoprost-induced reduction in tissue stiffness and cornea microstructure changes	Bimatoprost *	Cornea	Wang et al. [246]
Scheimflug-aided analysis showed a longitudinal reduction in CCD after bimatoprost use	Bimatoprost *	Cornea cell density (CCD)	Eraslan et al. [247]
Bimatoprost demonstrated cytotoxicity to tested cells	Lumigan 0.01%, and Lumigan 0.03%	Corneal endothelial cells	Tong et al. [248]
Bimatoprost was found to cause cell necrosis and stifle proliferation	Bimatoprost *	Corneal epithelium	Pozarowska et al. [249]
Bimatoprost 0.03% triggered accommodative spasm and the patient manifested pseudomyopia	bimatoprost 0.03% *	Accommodative system	Padhy and Rao [250]
Cystoid macula edema resulted after bimatoprost therapy	Lumigan 0.03%	Macula	Agange and Mosaed [251]
Prostaglandin use postoperatively associated with cystoid macular edema	Bimatoprost *	Macula	Wendel et al. [252]
A geriatric patient was found to have developed bilateral macula edema after receiving bimatoprost implants	Bimatoprost implant (Durysta)	Macula	Patel et al. [253]
Bimatoprost-induced inhibition of platelet activation action.	Lumigan UD^®^ (bimatoprost 0.3 mg/mL	Platelets	Moschos et al. [254]
Serous retinal detachment occurred after starting the bimatoprost regimen	Bimatoprost *	Retina	Addison et al. [255]
Bimatoprost 0.03 and 0.01 both resulted in conjunctival congestion after topical administration	Bimatoprost 0.01%	Scleral blood vessels	Ogundele et al. [256]
A case report of bimatoprost inducing late onset choroidal detachment in a patient after trabeculectomy	Bimatoprost 0.03%	Choroid	Nakakura et al. [257]
Bimatoprost 0.03% was associated with the thickening of the choroid.	Bimatoprost 0.03% *	Choroidal layer	Akyol et al. [258]
In-vitro analysis of the effect of bimatoprost on lamina cribosa astrocytes revealed apoptosis	Bimatoprost Acid *	Optic nerve head astrocytes	Shin et al. [259]
Bimatoprost resulted in prolonged vasodilation of ocular vessels, an indication of hyperemia	Bimatoprost 0.01% or Bimatoprost 0.03% *	Pig eyes in-vivo	Ogundele et al. [260]

* Formulation not completely stated.

## Data Availability

Data sharing is not applicable.
